# Effects of a fixed-intensity of endurance training and pistacia atlantica supplementation on ATP-binding cassette G4 expression

**DOI:** 10.1186/1749-8546-8-23

**Published:** 2013-11-25

**Authors:** Abbass Ghanbari-Niaki, Saleh Rahmati-Ahmadabad

**Affiliations:** 1 Department of Physical Education and Sport Science, University of Mazandaran, Baboulsar, Iran

## Abstract

**Background:**

Adenosine triphosphate-cassette binding protein (ABC) type G is considered as a part of reverse cholesterol transport (RCT) process in modification and metabolism of plasma and tissue cholesterol. This study aims to evaluate the effect of endurance training with or without Pistacia atlantica (Baneh) supplementation on the female rat tissues ABC type G expression and its correlation with plasma high-density lipoprotein cholesterol (HDL-C) concentration.

**Methods:**

Twenty Wistar rats (six to eight weeks old, 125–135 g weight) were arbitrarily allocated into training (n = 10) and control (n = 10) groups and further divided into saline-control (n = 5), saline-training (n = 5), Baneh-control (n = 5), and Baneh-training (n = 5). The training groups were given exercise on a motor-driven treadmill at 25 m/min (0% grade) for 60 min/day, 5 days/week for eight weeks. The rats were fed orally with Baneh extract and saline for six weeks. Seventy-two hours after the last training session, the rats were sacrificed and their tissues were excised for tissues ABCG4 expression which was detected by Real-time PCR method.

**Results:**

The ABCG4 gene expressions were significantly higher in liver (*P* = 02), small intestine (*P* = 06), and visceral fat tissues (*P* = 04) of the trained rats compared to the tissues of the control rats, but were lower in Baneh treated rats (liver *P* = 045, small intestine *P* = 06 and visceral fat *P* = 004) with lower HDL-C concentrations (*P* = 008).

**Conclusions:**

The Baneh administration lowered tissues ABCG4 expression and plasma HDL-C concentrations while endurance training increased the expression in female rat tissues.

## Background

Reverse cholesterol transport (RCT) is a process by which the excess cholesterol from peripheral tissues is returned to the liver where it is broken down and excreted [1,2]. This process prevents the macrophages with cholesterol to adhere the lining cell and arterials [3]. The Adenosine triphosphate-cassette binding protein (ABC) family is divided into seven categories from A to G based on their sequence and arrangement [4]. ABCG is recognized as a lipid transporter, and has several members including ABCG 1, ABCG 2, ABCG 3, ABCG 4, ABCG 5, ABCG 8, ABCG 11, ABCG 12, and ABCG 26 [5-8]. All the ABCGs except G2, play an important role in the RCT process [9]. Recently, ABCG1 has been recognized as one element of RCT in macrophage [10-12], ABCG4 transported cholesterol from macrophages to liver [9,10]. Both of ABCG1 and ABCG4 regulated cholesterol hemostasis in the brain [13]. ABCG4 is closely related to ABCG1 with 74% identity, 81% similarity at the amino acid level, and they are much more closely related to each other than to any other ABCG family members [9,14,15]. It is argued that like ABCG1, ABCG4 is also playing an important role in the cellular cholesterol efflux to HDL in the brain and eyes due to their higher ABCG4 expression [11,14,16,17]. Koshiba *et al*. [18] identified the expression of ABCG4 in different tissues such as testes, spleen, bone marrow, liver, and heart.

The ABCG subfamily members are classified as half-transporters which function as homo- or heterodimeric complexes [19]. The cholesterol-free high fat /atherogenic diets and unsaturated fatty acids administration suppressed ABCA1, ABCG1, ABCG5/ ABCG8 mRNA expression at least in small intestine and liver tissues [20-22], while exercise training at low to moderate intensity for moderate-term (more than 45 min/session) and long-term (90–120 min/session) could increase fatty acids mobilization and oxidation from adipose tissues and other fatty acid sources [23,24]. Exercise training along with a high dose of Baneh extract may provide a similar circumstance as high fat or athrogenic with endurance exercise training could do which has been use as a tool in some researches [23-26]. It is believed that macrophage-to-feces reverse cholesterol transport (RCT) is reflecting completed RCT [27], but it has no much significant impact on whole RCT which is completed by liver and small intestine on RCT and HDL biogenesis [27]. In this regards, Meissner *et al*. [27] showed that the voluntary exercise (10.2 ± 2.2 km, average speed: 1.78 ± 0.18 km/h) did not change plasma lipoprotein levels *in vivo* but changed the cholesterol efflux in running rats *in vitro*, and macrophage-to-feces RCT and the levels of ABCG5/ABCG8 gene expression remained unchanged. A high dose of Baneh extract was chosen because our GC-MS data analysis of Pistacia atlantica (Baneh) extract revealed that the percentage of trans isomer of oleic acid (elaidic acid) was higher than cis-isomer of oleic acid, similar to those reported elsewhere [28-30]. The Pistacia Chinensis Bunge (Chinese Pistache in English, and *huángliánmù* in Chinese) is a small to medium-sized tree, which is native to china, has bioenergic properties [31]. However, the extract of Pistacia Chinensis Bunge leaves by GC-MS contained 99 chemical compounds mainly terpenoides, which were considered as palmitic acid aromatic alcohol [32]. Long-sheng *et al*. [33] demonstrated that the average oil content of the fruit, flesh and kernel was 29.61%-38.61%, 40.38%-64.54%, and 44.81%-55.97%, respectively. Seven fatty acids are detected from the seed oil of P. chinensis, including palmitoleic acid, oleic acid, linoleic acid, linolenic acid, palmitic acid, stearic acid and arachidic acid, in which the total relative content of unsaturated fatty acids is up to 73.97%-87.41% [33]. *Pistacia Chinensis* methanolic extract (PCME) displayed broad inhibitory effects on platelet aggregation, calcium mobilization, ATP release, fibrinogen binding, and enhancement on cAMP production in unstimulated platelets diarrhea, dysentery, sore throat, cancer sore in mouth, carbuncles, and furuncles [34,35]. Research showed some similarities between the Pistacia atlantica, which is growing in different parts of Iran and Pistacia Chinensis Bunge [29-33]. It is likely that this type of Pistacia can be used for nutritional and medicinal purposes. However, the effects of Baneh extract on genes involved in RCT process and HDL biogenesis is barely known. Sobolova *et al*. [36] reported that silymarin has a positive feedback on ABC Transporters. On the other hand, exercise training as a metabolic stressor, particularly at low to moderate intensities even with a high-fat diet can increase fat oxidation and thereby spare muscle glycogen [23-27]. The responses of some of the ABC subfamily members such as ABCA1, ABCG1, and ABCG5/ABCG8 to acute and chronic exercise training have been investigated [20,37-42]. Most of these studies have reported a significant increase in ABCA1 and ABCG1 and ABCG5/ABCG8 human peripheral blood lymphocyte and rat tissues following an exercise training program. The effect of exercise as a metabolic stressor and Pistacia atlantica crud extract as a representative of high fatty acids content material on ABCG4 gene expression has not been studied. To the best of our knowledge, there is no information about the effect of exercise associated with the administration of Pistacia atlantica (Baneh) extract on tissue ABCG4 expression.

Based on the findings of studies reviewed above, a cholesterol–free, high fat diet/athrogenic diet, and unsaturated fatty acids in trans forms rather than a standard diet could suppress ABCA1, ABCG1, ABCG5-ABCG8 mRNA expression in rat tissues. It has been suggested that ATP-binding cassette (ABC) G subfamily of transporters are half-transporters have to homo- or heterodimerize in order to form functionally active transporters and there is very high similarity between ABCG4 and ABCG1 on the basis of their homology, regulation by oxysterol and retinoids, and amino acid levels [14-16,19]. Considering to these similarity between ABCG1 and ABCG4 and suppression of ABCG5, ABCG8, and ABCG1 by a high fat diet [20-22], we hypothesized that with these similarity between ABCG1 and ABCG4, and a higher trans-isomer oleic acid (elaidic acid) in Pistacia atlantica (Baneh) extract (Our GC-MS data) might has suppressive impact on ABCG4 expression in rat tissues. Furthermore, exercise training which is believed to increase fat mobilization and oxidation restores the action of a given Baneh extract. This study aims to investigate the effects of endurance training (8 weeks, 25 m/min, 60 min/session) and Pistacia atlantica (Baneh) extract on the liver, small intestine, visceral fat and kidney ABCG4 gene expression, and changes in ABCG4 mRNA expression that are as a result of changes in plasma HDL-C concentrations in female rats.

## Methods

### Herb material

The ripped fruit samples of Pistacia atlantica (Baneh) were collected from the fields of Maybod in Yazd province of Iran, and were stored at -18°C until use. Plant material was identified by herbarium collection in the Department of Biology, Faculty of Sciences, Mazandarn University, Iran.

### Preparation of the extracts

The extract was prepared according to the Hamdan *et al*. [43]. The whole ripped and dried fruits of Pistacia atlantica (Baneh) (10 g) were powdered and mixed with 150 mL of tap water, boiled for 45 min and cooled at room temperature. The distilled water was not used on the basis of herbalist’s recommendation. After that, the mixture was filtered twice by a Whatman filter (No. 4 filter, Whatman Company, England). The filtered solution volume was increased to 100 mL with tap water so that 1 mL was equivalent to 100 mg of the starting material [43]. The fresh extract was orally given at dose 100 mg/kg (7.5 μL/g of body weight) immediately at the end of the training session for six weeks. The control groups received the same volume of the extract following the same procedure.

### Preparation of the GC/MS analyses

The whole ripped and dried fruits of Pistacia atlantica were grounded in house electronic grinder (Moulinex, type-320, code-223, Made in France) to a fine powder. A part of the powdered plant was macerated with n-hexane (Merck Co., USA) for 72 h at room temperature, extracted by soxhlet extractor (Schott Duran, Germany, helped by faculty of Chemistry, University of Mazandaran) and evaporated by a rotary evaporator (Heiholph Instruments. D-91126 Schwabach. Type: Heizbad WB eco, Ser.No: 060819780, Germany). Chromatographic analysis was carried out on Hewlett Packard (HP) devices, 6890 series GC-MS apparatus combined only with a front detector FID and with two capillary Columns (Capillary Column 1:Model Number: HP 19091S-633- HP-1MS, and Capillary Column2: Model Number: Agilent 19091 J-133-HP-5) [Agilent Technologies, Inc. 2850 Centerville Road Wilmington, DE 19808 1-800-227-9770. Canada]. The fatty acid components of the Pistacia atlantica extracts were determined by library search software from The Wiley/NBS Registry Mass Spectral Data and in-house “BASER Library of Fatty Acid Constituents”.

### Animals

All experiments animals were conducted according to the policy of the Iranian convention for the protection of vertebrate animals used for experimental and other scientific purposes; and the protocol was approved by the Ethics Committee of the Sciences, University of Mazandaran (UMZ) and Babol University of Medical Sciences (BUMS, Mazandaran, Iran). Twenty Wistar female rats (6–8 weeks old; 125–135 g) were acquired from Pasteur’s Institute (Amol, Mazandaran) and maintained in the Central Animal House of Faculty of Physical Education and Sports Science of UMZ. Five rats were housed per cage (46-L volume) with a 12 h/12 h light/dark cycle. Temperature and humidity were maintained at 20.6°C - 23.4°C and 51.6% - 59.6%, respectively. The rats had free access to diets (a pellet form) and water. The rats were arbitrarily allocated into control (n = 10) and training (n = 10) groups. Rats were further divided into saline-control (SC, n = 5), saline-training (ST, n = 5), and Baneh-control (BC, n = 5), and Baneh-training (BT, n = 5) groups. The control group remained sedentary, whereas the training group underwent a moderate intensity running exercise program.

### Exercise training protocol

Training program began with familiarization of the rats with the apparatus for 4 days by placing them on the motor-driven treadmill. Training group was given exercise training for 5 days/week for 8 weeks [38,41]. The length of running time on the treadmill was progressively increased, from 10 m/min for 10 min/session to 25 m/min for 60 min/day, during the first 3 weeks. The rats continued to exercise 5 days per week for 60 min per day for the 5 weeks training period. The animals were scarified 72 h after the last exercise session. The food, but not the water, was removed from the rat cages 4 h before the sacrifices. The estrous cycle was determined in intact female rats by taking vaginal smears each morning by vaginal lavage. Smears were analyzed under a microscope to determine the type of cells present and the stage of the estrous cycle [44,45]. Only female rats showing at least two consecutive 4- or 5-day estrous cycles were used. The established estrous cycle in each female was used to select the day of the experiment, at which time the estrous cycle stage was confirmed by vaginal smear.

### Tissue biopsies

Seventy-two hours after the last training session, rats were anesthetized with intra peritoneal administration of a mixture of ketamine (supplied by Iranian company: Shiraz Iman Saba, Made in Holland) (30 – 50 mg/kg body weight) and xylazine (supplied by Iranian company: Shiraz Iman Saba, Made in Holland) (3 – 5 mg/kg body weight). Liver, small intestine, kidney, and visceral fat tissues were excised, cleaned, divided into two pieces, washed in ice-cold saline, and immediately frozen in liquid nitrogen and stored at -80°C until RNA extraction. Blood was collected in EDAT test tubes as anticoagulant and immediately processed for plasma preparation during 10 min centrifugation at 1000 × *g* and Plasma was also stored at -80 C for future analysis.

### RNA isolation, cDNA synthesis and Real-time PCR

Total RNA was extracted from 80 to 100 mg of tissue by RNA purification kits (AccuZol, Bioneer, Cat. No: k3090, Korea, supplied by Iranian company Ziest-takapoo, Tehran-Iran) Complementary DNA (cDNA) was extended from oligo-(dt)_18_ primers (0.25 μg per reaction) using cDNA synthesis kit (AccuPower RT PreMix) according to the manufacturer’s instructions. Real-time PCR was performed on light Cycler apparatus, (Corbet, Made in Australia). Real-time quantitative PCR was performed by QuantiFast SYBR Green PCR Kit (Cat. No. 204052; Qiagen, GmbH, Germany) in using 15 μL reaction containing 0.5 μL single-strand cDNA, 7.5 μL Master Mix, 1 μL of the each forward and reverse primers (5 pmol/μL), and 5 μL dH2O in a final reaction volume of 15 μL. ABCG4 sense primer was 5^′^-CCGAGACCAGCCGCTTC-^′^3, and antisense primer was ^′^5-TCCCAAAGACTGGGCAACTAAG-^′^3 (NM_138955, 71 bp) [46]. The β-actin sense and antisense primers were ^′^5-TATCGGCAATGAGCGGTTCC-^′^3 and ^′^5- CACTGTGTTGGCATAGAGG-3^′^ (NM_031144, 145 bp), respectively, which were used as normalizer gene.

### Plasma high density lipoprotein measurement

Plasma high density lipoprotein cholesterol (HDL-C) was determined by direct Immuno method (HDL-C Immuno FS, Pars Azmoun, Tehran, Iran). The Intra-assay coefficient of variation and sensitivity of the method were 1.2% and 0.03 mmol/L, respectively.

### Statistical analyses

The data were analyzed by the comparative threshold cycle method (CT). CT for each sample was determined by Rotor-Gene 3000 Software designed by Corbett Research, Australia. Δ-CT value was calculated by taking the CT of the ABCG4 gene and subtracting it from CT of β-actin. The ΔΔ-CT was calculated by subtracting the Δ-CT (sample) values from the Δ-CT (control). The relative quantification was calculated by the expression 2^-ΔΔCT^[40]. The Kolmogorov-Smirnov test was used to determine the normality of the distribution, and variables were found to be normally distributed. All results were expressed as means ± standard deviation (SD). All data were analyzed using a Two-way ANOVA (training × solution) and statistical significance was accepted at *P* < 0.05. Significant effects were followed by least significant difference post *hoc* test. Correlation was calculated by the Pearson Product Moment correlation. The repeated measures ANOVA were used to compare the rats’ body weight status at different weeks. All statistical analyses were performed with SPSS (Version 13; SPSS, Chicago, IL, USA).

## Results

**Figure 1:**
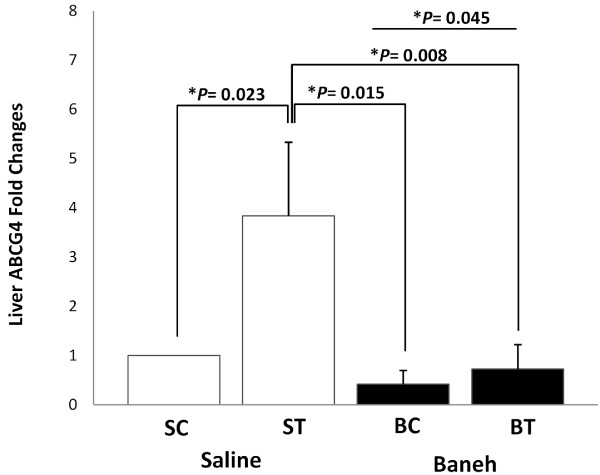
**The Real**-**time PCR of liver ABCG4 relative mRNA expression of saline**-**control (SC), saline-trained (ST), Baneh-control (BC), and Baneh**-**trained (BT) groups.** The results are expressed as mean ± SD. Each column is for five rats per group.

**Figure 2:**
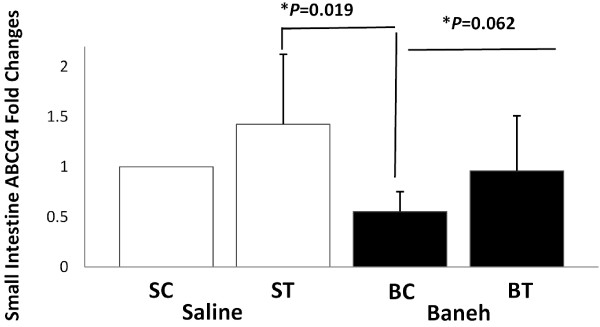
**The Real**-**time PCR of small intestine ABCG4 relative mRNA expression of saline**-**control (SC), saline-trained (ST), Baneh-control (BC), and Baneh**-**trained (BT) groups.** The results are expressed as mean ± SD. Each column is for five rats per group.

**Figure 3:**
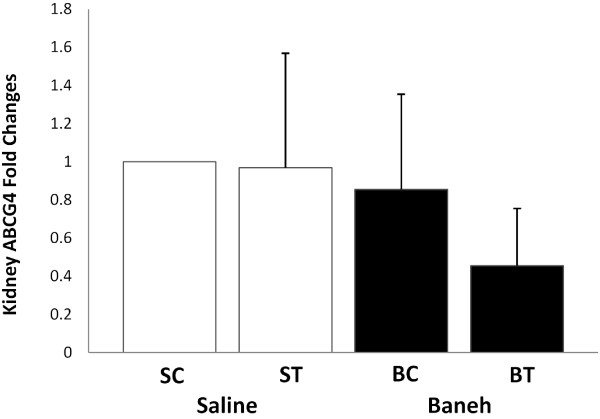
**The Real-time PCR of kidney ABCG4 relative mRNA expression of saline-control (SC), saline-trained (ST), Baneh-control (BC), and Baneh**-**trained (BT) groups.** The results are expressed mean ± SD. Each column is for five rats per group.

**Figure 4:**
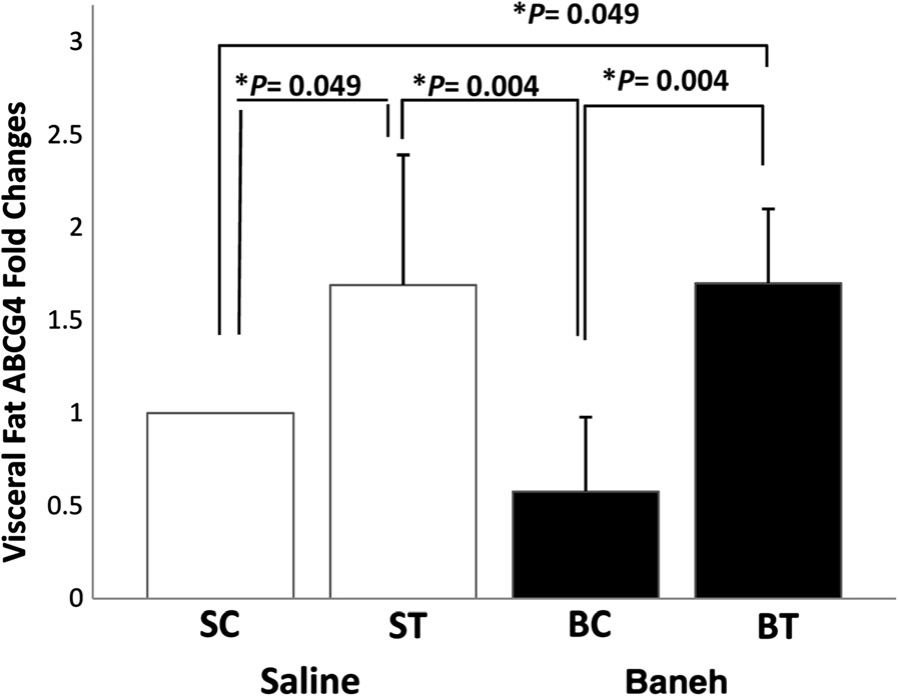
**The Real-time PCR of visceral fat tissue ABCG4 relative mRNA expression of saline-control (SC), saline-trained (ST), Baneh-control (BC), and Baneh**-**trained (BT) groups.** The results are expressed as mean ± SD. Each column is for five rats per group.

The GC-MS data analysis showed that the main components of Pistacia atlantica (Baneh) extract were oleic acid (C18:1; 9-trans-octadecenoic acid/Elaidic acid) (49.28%), trans hexadecanoic acid (C16:0, palmetic acid) (28.86%), hexadecenoic acid (palmitoleic acid (C16:1 n-7) (7.52%), Octadecanoic acid (C18:0; stearic acid) (3.87), Phenol, 4-(2-aminoethyl) (tyramine a monoamine) (2.69%), and Phenol, 3-pentadecyl-(3-N-pentadecylphenol) (1.58%) (Table 1). A significant change was observed in the liver ABCG4 mRNA expression (F = 4.667, *P* = 0.033). The following post *hoc* test showed that the expression of ABCG4 was significantly higher in saline-trained group than in SC, BC and BT (*P* = 0.023, 0.015, 0.008, respectively) groups. There was a significant difference between saline and Baneh treated animals (*P* = 0.045). The rats treated with Baneh had lower and significant ABCG4 mRNA expression (*P* = 0.045) (Figure 1). A significant change was observed in the small intestine ABCG4 expression (F = 3.65, *P* = 0.040). The following Post hoc test showed that there was a significant difference between ST and BC groups (*P* = 0.019). In addition, the difference between both treatments was significant (*P* = 0.062). Baneh induced suppression on small intestine ABCG4 mRNA which was to some extent restored by exercise training (Figure 2). There was no significant change in kidney ABCG4 mRNA expression (F = 0.83, *P* = 0.49) (Figure 3). The changes in visceral fat ABCG4 mRNA expression was significant (F = 5.61, *P* = 0.008). The results of the following post *hoc* test showed that the levels of visceral fat ABCG4 mRNA expression were significantly higher in both saline and Baneh-trained rats (*P* = 0.049 and *P* = 0.004, respectively) than in their control groups (Figure 4). There were no significant differences between saline-trained and Baneh-trained groups (*P* = 0.97) and also between both saline and Baneh control rats (*P* = 0.21). There was a significant difference between saline-trained rats and Baneh-control rats (*P* = 0.004) and between Baneh-trained and saline-control groups (*P* = 0.049) (Figure 4).

**Table 1:** **The main components of the whole fruits of Pistacia atlantica** (**Baneh**) **extracted by GC**-**MS analysis**

**RT [min.]**	**Library/ ID**	**Area%**	**Ref**	**Qual**
39.200	Elaidic acid (trans-9-Octadecenoic acid) (E-Oleic acid)	49.28	228773	99
36.056	Palmitinic acid	28.86	195439	99
35.196	Hexadecenoic acid	7.52	192904	97
	(Oleic acid ester)		(245468)	87
39.452	Stearic acid	3.87	231330	99
43.967	Tyramine	2.69	32747	38
44.258	3-pentadecyl-Phenol (Anacardol)	2.42	254859	83
8.079	Alpha-pinene	0.71	32180	96

**Figure 5:**
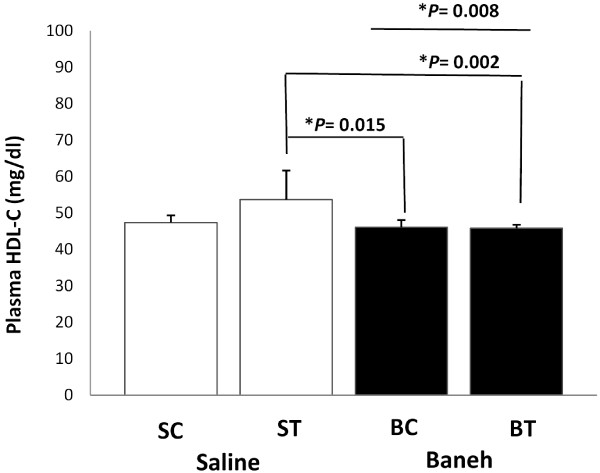
**Plasma HDL-C concentrations of saline-control (SC), saline-trained (ST), Baneh-control (BC), and Baneh-trained (BT) groups.** The results are expressed as mean ± SD. Each column is for five rats per group.

The changes in plasma HDL-C concentrations were significantly different between the groups (F = 4.33, *P* = 0.014). The saline-trained rat had higher and more significant plasma HDL-C than Baneh-control and trained groups (*P* = 0.015 and *P* = 0.002) (Figure 5). A significant difference was observed between saline and Baneh treatment (*P* = 0.008). The levels of plasma HDL-C were significantly lower in Baneh than in saline group (*P* = 0.008) (Figure 5). Positive and significant correlations were observed between plasma HDL-c concentrations and liver (r = 0.67, *P* = 0.001) and small intestine ABCG4 mRNA expression (r = 0.43, P = 0.056) (Table 2).

**Table 2:** **The correlations between plasma HDL**-**C concentrations and tissues ABCG4 mRNA expression**

**Correlation**	**Liver**	**Small intestine**	**Visceral fat**	**Kidney**
***r *** **value**	0.687	0.434	0.086	-0.173
***P *** **value**	0.01	0.056	0.717	0.478

The Repeated Measures ANOVA showed a significant difference in body weight changes inside each group (F = 10.78, *P* = 0.001) but not between different groups (F = 0.76, *P* = 0.52) (Table 3).

**Table 3:** **Body weight changes** (**g**) **of saline**-**control** (**SC**), **saline**-**trained** (**ST**), **Baneh**-**control** (**BC**), **and Baneh**-**trained** (**BT**) **groups during 8 weeks of the study**

**Week group**	**W1**	**W2**	**W3**	**W4**	**W5**	**W6**	**W7**	**W8**
**SC**	156.34 ± 10.91	168.8 ± 11.69	181.66 ± 13.32	194.85 ± 15.23	209.1 ± 17.36	215.32 ± 18.32	221.46 ± 20.76	227.6 ± 19.03
**ST**	153.54 ± 10.10	160.5 ± 11.20	152.86 ± 7.14	167.68 ± 6.02	189.1 ± 4.20	211.46 ± 3.91	215.72 ± 3.72	221.5 ± 3.70
**BC**	154.66 ± 23.45	166.5 ± 10.31	187.98 ± 8.80	199.5 ± 6.38	207.3 ± 4.96	210.36 ± 3.95	216.98 ± 2.73	221.4 ± 3.94
**BT**	159.14 ± 9.33	175.3 ± 25.79	190.38 ± 26.40	195.48 ± 25.08	200.9 ± 25.14	204.26 ± 24.55	208.68 ± 24.41	212.3 ± 25.01

The results were expressed as mean ± *SD*.

## Discussion

As a member of ABCG subfamily, ABCG4 was expressed in liver, small intestine, kidney, and visceral fat tissues, which exercise training increased ABCG4 mRNA expression in saline-treated liver, small intestine, visceral fat tissues but had no significant effect on saline–treated and trained kidney. The administration of Pistacia atlantica extract (Baneh) at a given dose (100 mg/kg) could suppress ABCG4 expression at a lower degree in kidney to a greater degree in liver tissue compared to the control tissues, while exercise training program could restore a Baneh-induced suppression of ABCG4 mRNA expression from a lower degree in liver to a higher degree in visceral fat and small intestine tissues, reinforce the effect of Baneh on ABCG4 mRNA expression in kidney. A Change in ABCG4 mRNA expression was accompanied by a significant change in plasma HDL-C concentrations, and there were significant correlations between plasma HDL-C changes and liver and small intestine ABCG4 mRNA expression changes. To our knowledge, this was the first study demonstrating the alterations of ABCG4 expression in response to exercise training and Baneh administration, and suggesting that ABC type G was involved in cellular cholesterol efflux and its homeostasis.

Like ABCG1, ABCG4 is involved in cholesterol efflux to HDL in the nervous system, particularly in the brain [10,11,13,17]. Wang *et al*. [10] reported that transient transfection with ABCG1 or ABCG4 stimulated isotopic cholesterol efflux to both HDL-2 and HDL-3, and HDL specific cholesterol efflux was approximately doubled for HDL-3 whereas efflux to HDL-2 was increased by approximate 50% in mouse macrophage. They also observed that the combination of ABCG1 and ABCG4 resulted in a small increase in cholesterol efflux. In the earlier reports, the transcript of the ABCG4 gene was detected in various tissues of the adult mouse, including brain, spleen, bone marrow, eyes, smooth muscle, and stomach but the expression was higher in brain, spleen, and eyes [14,47]. The expressions of ABCG4 were also reported in human adult tissues including brain, spleen, liver, thymus, testis, ovary, and small intestine [47]. Our results confirmed the previous findings and indicated that ABCG4 was also expressed in rat liver, kidney and visceral fat tissues, and the ABCG4 expression levels were lower in both control and trained Baneh treated tissues compared to their counterpart tissues treated by saline. Our GC-MS data analysis suggested that a lower ABCG4 expression in Baneh-treated tissues might be attributed to a higher content of unsaturated fatty acids, particularly elaidic acid in Baneh extract, which were partially in agreement with the previous reports [20,22,37,48]. de Vogel-dan den Bouch *et al*. [22] found that a cholesterol-free, high fat diet (C18:1, C18:2, and C18:3) down regulated cholesterol transporter gene in mice’s small intestine and decreased in ABCA1, ABCG5, and ABCG8 gene expression in mice’s small intestine. Vecera *et al*. [48] demonstrated that a high cholesterol diet reduced liver ABCG5 and ABCG8 gene expression but the administration of silymarin (1% and 3%) somewhat restored the suppression of ABCG expression induced by a high cholesterol diet in rats. Ghanbari-Niaki *et al*. [37] found that giving a high dose of aqueous extract of Pistacia atlantica (Baneh) reduced small intestine and increased kidney ABCG8 expression. Côte *et al*. [20] reported that an atherogenic diet decreased liver ABCG8 but increased ABCG5 mRNA expression, and found lower levels of ABCG5 and ABCG8 mRNA expression in rat’s small intestine.

The mechanism (s) of Baneh extract in influencing the expression of ABCG4 in female rat tissues was poorly understood. The farnesoid X receptor as a nuclear receptor was down-regulated by an atherogenic diet in rat’s liver but not in intestine (Jejunum) [20]. Uehara *et al*. [49] reported that unsaturated fatty acids suppress ABCG1 and ABCA1 genes by a mechanism which involves the binding of LXR/RXR to the promoters. The impact of different types of physical exercise/training on ABCA1, ABCG1, ABCG5, and ABCG8 mRNA expression in human leukocytes (lymphocyte), liver, small intestine, heart, and gastrocnemius tissues was studied [20,37-42]. Our result in examining the impact of exercise training and Baneh extract on ABCG4 gene expression was in agreement with those of other studies that focused on ABCG1, ABCG5, and ABCG8 mRNA expression. Ghanbari-Niaki [37] reported that the small intestine and kidney ABCG8 mRNA were expressed differently by exercise training program (25 m/min, 60 min/session, 5 day/week, and for 8 weeks) and Baneh administration. They also mentioned that in contrast to small intestine, the level of kidney ABCG4mRNA was not suppressed by the administration of Baneh extract, but it was reduced at the end of exercise training program. Côté *et al*. [20] reported that the expression of liver ABCG8 mRNA but not ABCG5 mRNA expression increased in response to exercise and decreased following an atherogenic diet. They also reported that both ABCG5 and ABCG8 mRNA expression increased rats feeding by a standard not atherogenic diets.

The changes in tissues ABCG4 mRNA expressions were accompanied by higher plasma HDL-C concentration in saline-trained rats. A significant correlation was found between liver and small intestine ABCG4 fold changes and plasma HDL-C concentrations. Although the mechanism (s) in endurance training influencing the ABCG4 mRNA expression in female rat tissues was not clearly understood, the alterations in ABCG4mR expression might be attributed to peroxisome proliferator-activated receptor (PPAR), liver X receptor (LXR), and farnesoid X receptor (FXR) [20,50,51]. Butcher *et al*. [39] indicated that LXR α, PPAR α and PPAR γ were significantly increased following an 8-week of low-intensity exercise program. Côté *et al*. [20] reported that feeding atherogenic diet reduced liver FXR (by about 70%) but did not change liver LXR gene expression and FXR expression in small intestine. Zhang *et al*. [52] demonstrated that swimming exercise training (5 day/week for 3 months) significantly increased PPARα no PPARγ gene expression in OLETF rat liver. As shown by Horowitz *et al*. [53], endurance training (70-85% HRmax, 35–45 min/session, 4 days/week for 12–14 weeks) resulted in a twofold increase in mean skeletal muscle PPARα protein compared to untrained skeletal muscle. The secretion and maturation of HDL was complex and is not simply clarified by measuring just one ABCG subfamily member such as ABCG4 gene expression. However, dimerization is a requirement of becoming a functional transporter [8,9,19]. ABCG4 and ABCG1 could effect synergically on cellular cholesterol efflux to HDL by forming homo or heterodimer complex [10]. In this study, the exercise training enhanced the ABCG4 mRNA expression and restores it in saline and Baneh treated liver, small intestine, and visceral fat not kidney tissues, and indicated that ABCG4 was expressed at different magnitudes in response to exercise training program and Baneh administration, which might be due to the capacity and ability of each tissue to manipulate and dispose unsaturated fatty acids content of Baneh extract. Further studies are required to provide more information on the impact of different exercises with varying intensity and duration, along with Baneh extract on brain, skeletal muscles, heart, and spleen ABCG subfamily members gene expression and other involved mechanisms.

## Conclusions

The Baneh administration lowered tissues ABCG4 expression and plasma HDL-C concentrations while endurance training increased the expression in female rat tissues.

## Abbreviations

(ABC): Adenosine triphosphate-cassette binding protein; (ABCGs): ATP-binding cassette transporters G; (Baneh): Pistacia atlantica; (RCT): Reverse cholesterol transport; (HDL-C): High density lipoprotein cholesterol; (SC): Saline- control; (BC): Baneh- control; (ST): Saline- training; (BT): Baneh-training.

## Competing interests

The authors declare that they have no competing interests.

## Authors’ contributions

AGN conceived and designed this study. SRA collected the materials, performed the experiments, analyzed the data and wrote the manuscript. AGN revised and improved the quality of the paper. Both authors read and approved the final version of the manuscript.
